# Prelimbic cortex glucocorticoid receptors regulate the stress-mediated inhibition of pain contagion in male mice

**DOI:** 10.1038/s41386-020-00912-4

**Published:** 2020-11-23

**Authors:** Navdeep K. Lidhar, Soroush Darvish-Ghane, Sivaani Sivaselvachandran, Sana Khan, Fatima Wasif, Holly Turner, Meruba Sivaselvachandran, Neil M. Fournier, Loren J. Martin

**Affiliations:** 1 Department of Psychology, University of Toronto Mississauga, Mississauga, ON L5L 1C6 Canada; 2 Department of Cell and Systems Biology, University of Toronto Mississauga, Mississauga, ON L5L1C6 Canada; 3 Department of Psychology, Trent University, Peterborough, ON K9J 7B8 Canada

**Keywords:** Neuroscience, Empathy

## Abstract

Experiencing pain with a familiar individual can enhance one’s own pain sensitivity, a process known as pain contagion. When experiencing pain with an unfamiliar individual, pain contagion is suppressed in males by activating the endocrine stress response. Here, we coupled a histological investigation with pharmacological and behavioral experiments to identify enhanced glucocorticoid receptor activity in the prelimbic subdivision of the medial prefrontal cortex as a candidate mechanism for suppressing pain contagion in stranger mice. Acute inhibition of glucocorticoid receptors in the prelimbic cortex was sufficient to elicit pain contagion in strangers, while their activation prevented pain contagion in cagemate dyads. Slice physiology recordings revealed enhanced excitatory transmission in stranger mice, an effect that was reversed by pre-treating mice with the corticosterone synthesis inhibitor metyrapone. Following removal from dyadic testing, stranger mice displayed enhanced affective-motivational pain behaviors when placed on an inescapable thermal stimulus, which were reversed by metyrapone. Together, our data suggest that the prelimbic cortex may play an integral role in modulating pain behavior within a social context and provide novel evidence towards the neural mechanism underlying the prevention of pain contagion.

## Introduction

Emotional contagion—matching an emotional state between individuals—is a primitive form of empathy and serves as an important social cue in humans and animals [1–3]. In addition to distress [4], fear [5], and anxiety [6], pain is an emotionally contagious phenomenon [7, 8]. Pain contagion represents a sensory tuning process by which the social observation of a painful experience in another individual heightens one’s own pain sensitivity. In this respect, pain behaviors are highly salient and evolutionarily adaptive cues to inform others about distress within a social context and warn members about potentially dangerous situations [9]. The mere presence of another individual impacts the sufferer’s pain experience, with the specific audience playing a critical role. Human and rodent studies have shown that observing and experiencing pain with a familiar, but not unfamiliar (i.e., stranger) conspecific enhances pain behavior and sensitivity in both members of the dyad [7, 10, 11]. Moreover, stranger conspecifics’ social interaction causes the prevention of pain contagion by activating the endocrine stress response [12]. In mice, the lack of pain contagion is specific to male stranger dyads in which both mice received the pain stimulus. We suspect that the prevention of pain contagion in male mouse stranger dyads is related to the potential threat of an aggressive encounter with an unfamiliar male mouse since female–female aggression rarely occurs in group-housed laboratory mice [13].

The influence of social threat on pain sensitivity is a burgeoning field with perceived social threat level shown to modulate pain behavior in mice [14] and humans [15, 16]. In response to potentially threatening situations, a signaling cascade is initiated that culminates in the release of adrenal glucocorticoid hormones (mainly corticosterone (CORT) in animals and cortisol in humans). These glucocorticoid hormones exert their effects through glucocorticoid and mineralocorticoid receptors (GR and MR, respectively), with GRs predominately mediating the response to acute stress [17]. In the brain, GRs are present in both subcortical and cortical structures exhibiting high distribution in the medial prefrontal cortex (mPFC) [18, 19]. Following exposure to an acute stressor, corticosterone is elevated in the mPFC and remains elevated for up to 90 min [20]. Moreover, recent evidence points toward a role for the mPFC in behavioral responses to socially threatening [21] and stressful situations [22], while bilateral lesions of the mPFC abolish the social facilitation of pain [10]. Thus, the current study sought to determine whether the social threat of an unfamiliar mouse suppresses pain contagion through GR signaling in the mPFC. Also, we view the prevention of pain contagion in strangers as an outright suppression of pain behavior. This led us to explore the secondary hypothesis that detecting a social threat causes the inhibition of pain behavior in unfamiliar male mice, and this can lead to an enhancement of pain sensitivity once the social threat has been removed.

In the present study, we measured the expression of c-Fos, an immediate early gene product, and phosphorylated GR (p-GR) across multiple cortical brain regions in mice tested alone or with a stranger/cagemate following exposure to a nociceptive assay. Immunohistochemical analysis showed elevated c-Fos and p-GR activity in the mPFC of stranger dyads. Local infusion of RU-486, a GR antagonist, into the prelimbic cortex of stranger dyads increased pain behavior, while a local infusion of corticosterone, a GR agonist, decreased pain behavior in cagemate dyads. To determine whether synaptic transmission of cortical neurons in the prelimbic cortex was altered in stranger dyads, we measured spontaneous activity in layers II/III following pain testing in mice treated with or without metyrapone, a drug that blocks the synthesis of CORT. Finally, we also demonstrate that stranger and cagemate mice display different pain behaviors when removed from their dyadic condition, and tested alone on an inescapable thermal apparatus.

## Materials and methods

### Mice

Mice were bred in-house and consisted of young (6–12 weeks) adult male CD-1 mice. All mice were housed in non-ventilated cages in groups of 4–5, maintained in a temperature-controlled (20 ± 1 °C) environment with 12:12-h light: dark cycle (lights on at 7 am and off at 7 pm) with access to food (Harlan Teklad 8604) and water ad libitum. Experiments were conducted only during the light period. All procedures were performed in accordance with the guidelines of the Canadian Council on Animal Care and approved by the University of Toronto Animal Care Committee.

### Acetic acid test and social conditions

Pain behavior was assessed using the acetic acid abdominal constriction test, under three social conditions: (1) alone, (2) in a dyadic condition where mice were drawn from the same home cage (cagemates), or (3) in a dyadic condition where mice were drawn from different home cages (strangers). All testing occurred near mid-photoperiod (10 am–4 pm). At the beginning of the test day, mice were brought to the experimental room and left undisturbed for 1 h before testing began. Mice were then placed on a glass surface within red Plexiglas cylinders (30 cm high × 15 cm diameter) either alone or with a cagemate/stranger social partner and allowed to habituate to the cylinder for 30 min. Mice were then briefly removed and injected intraperitoneal with acetic acid (0.6% in physiological saline; 10 ml/kg). Mice were placed back in their cylinders and observed continuously for 30 min. Stereotypical abdominal constrictions or “writhes” (lengthwise constrictions of the torso with a concomitant concave arching of the back) were counted over this period, sampling for 5 s every 20 s for a total of 90 observations. The presence of a “writhe” within the 5 s observation period was considered a positive sample. The percentage of writhing behavior was calculated by dividing the number of positive samples by total samples and multiplying by 100 %. Sampling in this manner allows for a higher degree of accuracy and higher interrater reliability than counting constrictions [7]. In experiments where drug delivery was required, mice were pretreated systemically or via microinjection 30 min before acetic acid injection (i.e., at the beginning of habituation to the test cylinders). The synchronization of writhing was calculated using joint probability, a statistical measure that calculates the likelihood of two events occurring together and at the same point in time. Since probabilities are combined using multiplication, the “expected” joint probability of writhing was calculated by multiplying the writhing score of mouse #1 by the writhing score of mouse #2. The observed writhing sample for a mouse was then divided by the expected joint probability of its dyad to calculate the percentage of synchronized writhing above expected levels. In all experiments (behavioral, immunohistochemistry, and electrophysiological), both mice within a dyad were injected with acetic acid. Behavior was video recorded for offline analyses, and all experimenters were blind to social condition and drug condition being coded.

### Thermal nociceptive reflexes and affective-motivational behavior

To evaluate whether pain behaviors—other than writhing—were altered following social interactions or removal from dyadic pain testing, a subset of mice were placed on an inescapable thermal stimulus for 45 s at three different time points throughout the testing session. Briefly, mice were placed singly on a 52.5 °C hotplate for 45 s before any social interaction (baseline), following a 30 min social interaction with a cagemate or stranger (social-no pain) and following acetic acid dyadic testing (social-pain). Baseline responses were collected immediately following 1-h habituation to the test environment and before the social interaction/acetic acid test. The acetic acid dyadic testing was performed as described above, with each mouse receiving an acetic acid injection. In a subset of stranger dyads, metyrapone (50 mg/kg, s.c.) was injected 30 min before baseline testing. For these experiments, the same pair of mice were used for the social-no pain phase and then the social-pain phase. We categorized nociceptive reflexive (rapid flicking of the limb) and affective-motivational (paw licking, paw guarding, and escape jumping) pain behaviors over the entire 45 s duration of the trial based on previous studies [23–25]. The primary distinction is that rapid reflexive retraction of the paw occurs in response to nociceptive sensory information but ceases once the stimulus has been removed—these responses mainly involve spinal and brainstem circuits. In contrast, affective-motivational responses are temporally-delayed—relative to noxious stimulation—and characterized by hyperlocomotion, rearing, and escape behavior. Affective responses are complex and rely on limbic and cortical circuits in the brain, which may indicate motivation to reduce aversive simulation by licking tissue, protecting tissue, or attempting to escape [23]. Video files were coded using BORIS (Behavioral Observation Research Interactive Software, http://www.boris.unito.it). The number of cumulative reflexive responses were counted, while the cumulative duration of the affective-motivational responses was summed over the entire duration of the trial. The area under the curve (AUC) of the cumulative responses was calculated using the trapezoidal rule.

### Tissue preparation and immunohistochemistry

Ninety-minutes (for c-Fos) or 30-min (for GR phosphorylation) following acetic acid injection, a subset of mice, were deeply anesthetized with sodium pentobarbital (100 mg/kg, i.p.) and underwent transcardiac perfusion with 0.1 M phosphate-buffered saline (PBS, pH = 7.4) followed by 4% (w/v) buffered paraformaldehyde. The brains were extracted and post-fixed in the same fixative for 4 h at 4 °C before undergoing PBS sucrose infiltration (30% w/v sucrose) and sectioning on a cryostat (Cryostar NX50, ThermoFisher Scientific, Waltham, MA) at a section thickness of 40 µm. All serial sections were collected and stored at −20 °C in a cryoprotectant solution consisting of 30% (w/v) sucrose, 1% (w/v) polyvinylpyrrolidone, and 30% (v/v) ethylene glycol in 0.1 M PBS until use. Immunostaining for the immediate early gene product c-Fos or GR phosphorylation at Ser(211) was performed on free-floating coronal sections as described previously [26, 27].

#### c-Fos immunohistochemistry

All procedures were performed under gentle agitation and at room temperature unless noted otherwise. Sections were first washed several times in 0.1 M PBS (pH 7.4; 6 times for 10 min each). To minimize endogenous peroxidase activity, slices were incubated in a solution of 0.3% (v/v) H_2_O_2_/PBS for 30 min. The sections were then rinsed several times in 0.1 M PBS before being placed in a blocking buffer containing 5% (v/v) Normal Horse Sera, 1% (w/v) Bovine Serum Albumin, and 0.3% (v/v) Triton X-100 dissolved in 0.1 M PBS for 1 h. After blocking, sections were incubated with a primary anti-rabbit polyclonal c-Fos antibody (1:10,000, ABE457 Millipore) and dissolved in the same blocking buffer for 48 h at 4 °C, followed by incubation with a secondary biotinylated antibody (goat anti-rabbit, 1:500, 2 h, room temperature, Vector Laboratories) and then treatment with avidin-biotin-peroxidase complex (ABC, 1:500, 1 h, Vector Laboratories). After rinsing first in PBS then in 0.175 M sodium acetate (pH = 7.0), the antibody-peroxidase complex was visualized with a solution of 2.5% (w/v) nickel ammonium sulfate, 0.2% (w/v) 3,3-diaminobenzidine HCl, and 0.083% (v/v) H_2_O_2_ in aqueous 0.175 M sodium acetate to yield a blue-black reaction product. After approximately 15–20 min, the tissue was rinsed in the acetate solution, washed in PBS, and then placed into saline. The sections were mounted onto charged glass slides (Fisher SuperFrost Plus, Fisher Scientific) and left to air dry overnight. Slides were dehydrated through an ascending series of alcohols, cleared in xylene, and coverslipped with Entellan mounting medium.

#### Phosphorylated glucocorticoid receptor immunohistochemistry

Slices were rinsed with 1× PBS and then washed with PBS-T three times. Slices were then incubated with 4% normal donkey serum in PBS-T for 2 h at room temperature. Following three washes with PBS-T, slices were then transferred to 1:1000 solution of the anti-rabbit phosphorylated glucocorticoid receptor antibody (Cat. #4161, Cell Signaling Technology) for 48 h at 4 °C. After rinsing with PBS-T three times, slices were then incubated in anti-rabbit Cy3 secondary antibody (Jackson laboratories) for 2 h at room temperature. Slices were then mounted onto VWR SuperFrost slides, dried and dipped to rehydrate in a 1:50,000 DAPI solution. Slides were then coverslipped with an aqueous mounting medium.

### Image analysis and quantification

#### c-fos

Fos immunoreactivity was examined in the medial prefrontal cortex (infralimbic, prelimbic, and anterior cingulate), amygdala (basolateral and central amygdala), hippocampus (CA1, CA2/CA3, and dentate gyrus), and hypothalamic paraventricular nucleus with each region being delineated based on cytoarchitectonic boundaries found in the Mouse Brain Atlas (Franklin and Paxinos, 1997). Microscopic images of the regions of interest were photographed with a 1600 × 1200-megapixel digital camera (Optronics MicroFire) attached to a light microscope (Nikon Eclipse 80i). For each region, images were taken at 4x magnification to identify the regions and 20x magnification to quantify immunostaining. All photomicrographs were exported in TIFF format and processed in ImageJ (http://rsbweb.nih.gov/ij/; National Institutes of Health, USA). Images were converted to (8-bit) grayscale, and Fos^+^ immunolabeled cells were counted using the Particle Analyzer tool in ImageJ with the application of the same image, threshold, size, and circularity settings for each brain region. For each animal, cell counts were obtained from between three and four sections. Each region of interest was outlined and measured using ImageJ to calculate the area. The number of Fos^+^ cells for each region of interest was then expressed as cells per square millimeter.

#### Phosphorylated glucocorticoid receptor (p-GR)

The expression of p-GR in rostral ACC, prelimbic cortex, and anterior insular cortex across three brain slices per ROI were examined. Images were taken using the Cytation 5 Imaging Multi-Mode Reader (BioTek, Winooski, VT). Images were taken at 4× for regional identification and 20× for quantification. Using the Gen5 software cell counter tool, cells expressing p-GRs were counted using the same threshold, size, and circularity settings for each brain region. Each region of interest was outlined and measured using Gen5. The number of p-GR^+^ cells for each region of interest was expressed as cells per square millimeter.

### Plasma corticosterone

Following behavior, mice were sacrificed, and trunk blood was collected. Blood samples were kept on ice and centrifuged at 4 °C at 15,000 r.p.m. for 15 min. Plasma was extracted from the samples and frozen at −80 °C until processing. Corticosterone levels were computed using enzyme immunoassay (Cayman Chemical Company, Kit 500655). Samples and standards were assayed in duplicate at a 1:800 dilution according to the manufacturer’s protocol. Single absorbance readings for standards and samples were obtained at 405 nm using a Biotek Plate reader (Synergy HT, Winooski, VT). These values were used to calculate plasma corticosterone levels (ng/ml) based on linear regression of the standard curve using a log–logit transformation.

### Prelimbic and ACC microinfusions

Stainless steel injection guide cannulas (C315GS-5/spc, P1 Technologies, Roanoke, VA) were bilaterally implanted into the prelimbic cortex (AP1 2.33 mm, ML 0.40 mm, and DV 1.30 mm) or the anterior cingulate cortex (AP1 2.00 mm, ML 0.40 mm, and DV 1.15 mm) at 6 weeks of age, DV was measured relative to brain surface [28]. The internal cannulas extended 0.5 mm from the guide cannula. Mice were allowed to recover for 7–8 days, after which microinjections were done under slight isoflurane anesthesia, 30 min preceding acetic acid injection. The internal cannulas were connected to a microliter syringe (1700 series Hamilton syringe, 100 μl, Harvard Apparatus, Montreal, QC) and pump (Pump 11 Elite Nanomite, Harvard Apparatus, Montreal, QC) via calibrated tubing. To limit diffusion, RU-486 or corticosterone was infused in a volume of 0.25 μl per side over 5 min, after which the injectors were left in position for an additional 1 min.

#### Verification of cannulae placement

After experiments, animals were killed by decapitation, and brains were rapidly removed and post-fixed in 4% paraformaldehyde. Coronal sections (40 μm) were then cut at the level of the mPFC using the Cryostar NX50 and mounted onto VWR superfrost slides, nissl stained or dipped in DAPI, and placed under coverslips to verify probe placements.

### Drugs

Metyrapone (2-methyl-1,2-di-3-pyridyl-1-propanone), RU-486 (11β-(4-dimethyl-amino)-phenyl-17β-hydroxy-17-(1-propynyl)-estra-4,9-dien-3-one) and corticosterone were purchased from Sigma Aldrich (St. Louis, MO). Drug dose and pre-treatment timing were chosen based on our previous work [11], and pilot experiments to identify proper microinjection dosing parameters in which outright writhing behavior was not affected. Metyrapone was dissolved in saline and injected, 30 min before acetic acid injection, subcutaneously at a concentration of 50 mg/kg. Corticosterone was dissolved in aCSF to a concentration of 20 ng/μl. RU 486 was not dissolvable in aCSF or saline, and thus aliquots were prepared in DMSO (dimethyl sulfoxide) to obtain a final concentration of 2 μg/μl. In all dyadic conditions, both mice received the same drug treatment.

### Electrophysiology

Brain slices were prepared based on previous work [29]. Mice were anesthetized with 5% isoflurane and killed by decapitation. The brains were quickly removed and placed in cold (4 °C) oxygenated (95% O_2_; 5% CO_2_) artificial cerebrospinal fluid (aCSF) consisting of 124 mM NaCl, 4.4 mM KCl, 2 mM CaCl_2_, 1 mM MgSO_4_, 25 mM NaHCO_3_, 1 mM NaH_2_PO_4_, and 10 mM glucose. Brain slices (300 μm) containing coronal sections of the prelimbic cortex were prepared with a VT1200S tissue slicer (Leica, Concord, ON). Slices recovered for a minimum of 60 min in a submerged holding chamber (25 °C) before recording. Following recovery, slices were placed in a recording chamber where they were continuously perfused with oxygenated (95% O_2_; 5% CO_2_) aCSF at a rate of 2 ml per min. Whole-cell voltage-clamp recordings from layer II/III pyramidal neurons of the prelimbic cortex was obtained under visual guidance using a 40× objective on a Zeiss Axioskop FS upright microscope. Layer II/III was specifically targeted because p-GR expression was quantified in these layers, and synaptic transmission in response to an acute stressor is enhanced in layers II/III [29]. Recordings were made with electrodes (4–6 MΩ) fabricated using a horizontal puller (P1000; Sutter, Novato, CA) and filled with an internal solution containing, cesium methanesulfonate-based intracellular solution (120 mM Cs-MeSO_3_, 5 mM NaCl, 10 mM HEPES, 1 mM MgCl_2_, 0.5 mM EGTA, 2 mM MgATP, 0.1 mM Na3GTP, 10 mM HEPES, and pH 7.3, 285–290 mOsmol). QX314 (5 mM) was included in the intracellular solution to block postsynaptic sodium currents. Neurons were held at −60 mV, the reversal potential for chloride, to assess glutamatergic synaptic transmission. In the same cell, neurons were held at 0 mV, the reversal potential for sodium, to assess GABAergic synaptic transmission. Electrophysiological recordings were performed using an Axon 700B amplifier (Axon Instruments, Foster City, CA), low pass filtered at 1 kHz, and digitized at 10 kHz with Clamplex (version 10.6; Molecular Devices). Spontaneous synaptic currents (sEPSC or sIPSC) were detected using Mini Analysis v. 6.0.3 (Synaptosoft, Decatur, GA, USA) with a detection threshold set three times higher than the level of baseline noise. The accuracy of detection was visually confirmed for each event in each recording. Spontaneous currents were selected so that the rise and decay phases did not contain any overlapping events. For individual neurons, the amplitude and frequency were analyzed during 5-min bins. Given that the frequency of events (*Freq)* measures the probability of neurotransmitter release and the amplitude (*Amp*) measures the response of postsynaptic site to the released neurotransmitter, their product gives the synaptic driving force of the neurotransmitters involved [29, 30]. Thus, we calculated synaptic drive as previously reported [29] using the formula:

$${\mathrm{Synaptic}}\,{\mathrm{drive}}\left( {\frac{{{\mathrm{sEPSCFreq}}\,{\times}\,{\mathrm{sEPSCAmp}}}}{{{\mathrm{sIPSCFreq}}\,{\times}\,{\mathrm{sIPSCAmp}}}}} \right).$$

### Statistical analysis

All statistical analyses were conducted using SPSS v24 software. Behavioral data (percent abdominal constrictions) were analyzed using one-way or two-way ANOVAs, corrected for multiple comparisons, with between-subject factors of social context or drug. c-Fos (neurons/mm^2^) and p-GR labeling (neurons/mm^2^) were averaged for each animal across 2–3 tissue sections, and then group means calculated. In most experiments, the vehicle-treated alone, stranger, and cagemate conditions were analyzed using a one-way ANOVA to compare cagemate and stranger responses directly. In experiments where the effects of metyrapone were assessed, cagemates were not inputted into the two-way ANOVA because of the unbalanced experimental design. The thermal stimuli experiment was analyzed using a mixed two-way ANOVA with the between-subject factor of social context and the repeated measure factor of phase of testing (baseline, social-no pain, and social-pain). For all analyses, significance was set at *p* < 0.05. Tukey’s post-hoc tests were conducted; significant comparisons are only shown for analogous drug-treated groups between (i.e., saline-alone vs. saline-strangers) or within (i.e. saline-strangers vs. metyrapone-strangers) the social context.

## Results

### c-Fos expression following social interactions and pain

**Fig. 1:**
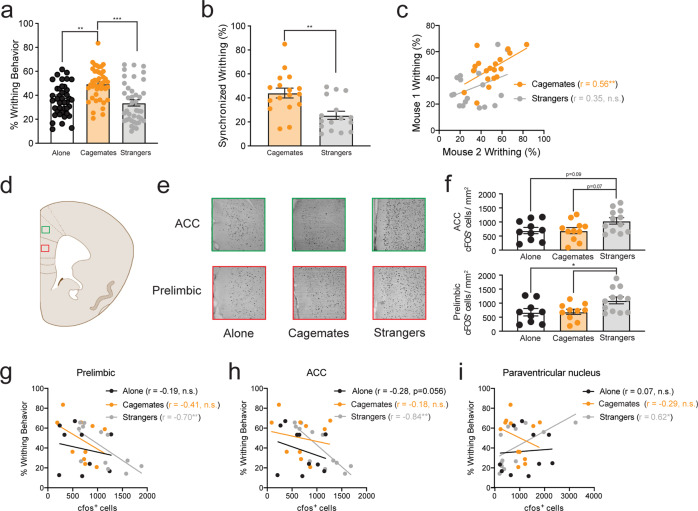
Social context enhances writhing behavior in familiar mice but increases c-Fos expression in the prelimbic and anterior cingulate cortex of stranger mice. **a** Enhanced writhing behavior in mice paired with cagemate (*n* = 39) compared with mice tested with a stranger (*n* = 40) or alone (*n* = 45) (mean ± SEM, one-way ANOVA, *F*_2,121_ = 10.87, *p* < 0.001). Sample sizes for each condition are larger than other experiments because mice were pooled from c-Fos (Fig. 1 and Supplementary Fig 2) and western blot experiments (Supplementary Fig. 3) **b** Higher co-occurrence of writhing behavior in cagemate dyads than stranger dyads. Using data from **a**, the expected number of samples with writhing in both mice of the dyad was calculated as a joint probability. Bars represent the mean ± SEM of episodes of joint writhing expressed as a percentage (independent samples *t*-test, *t*_31_ = 3.42, *p* < 0.01). **c** Writhing behavior is significantly correlated between mice within a dyad for cagemates (*r* = 0.56, *p* < 0.01), but not strangers (*r* = 0.35, *p* > 0.05). **d** Illustration showing the target areas analyzed for the prelimbic (red box) and anterior cingulate cortex (green box). **e** Representative images showing c-Fos staining in the prelimbic and anterior cingulate cortex for mice tested alone (*n* = 10) or within a cagemate (*n* = 11) or stranger (*n* = 12) dyad. **f** c-Fos expression (c-Fos+ cells/mm^2^) is increased in the prelimbic cortex (mean ± SEM, one-way ANOVA, *F*_2,31_ = 3.76, *p* < 0.05) and the anterior cingulate cortex (mean ± SEM, one-way ANOVA, *F*_2,31_ = 4.36, *p* values for post-hoc testing were trending toward significance and are indicated on the graph). Writhing behavior in the stranger, but not alone or cagemate conditions is significantly correlated with c-Fos expression in the **g** prelimbic (strangers: *r* = −0.70, *p* < 0.01; cagemates: *r* = −0.41, *p* > 0.05; alone: *r* = −0.19, *p* > 0.5), **h** anterior cingulate cortex (strangers: *r* = −0.844, *p* < 0.001; cagemates: *r* = *−0.19, p* > 0.05; alone: *r* = −0.28, *p* > 0.05) and **i** paraventricular hypothalamic nucleus (strangers: *r* = 0.618, *p* < 0.05; cagemates: *r* = −0.29, *p* > 0.05; alone: *r* = 0.07, *p* > 0.05). **p < 0.01, ***p < 0.001.

As shown in Fig. 1a, writhing behavior (i.e., % of positive samples with a “writhe”) in the dyadic condition increased significantly in cagemate, but not stranger pairs, replicating our previous findings [11]. To determine whether writhing behavior was synchronized, as described elsewhere [7], we calculated the joint probability of total writhing, expressed as a percentage of the group mean. In line with contagion effects, writhing co-occurred in time more in cagemate dyads than stranger dyads (Fig. 1b). Further, cagemate, but not stranger dyads, exhibited highly correlated writhing behavior during the abdominal constriction test (Fig. 1c). In general, observed behaviors other than writhing were similar across all conditions during social interactions. However, there was a higher incidence of hair-nibbling between stranger pairs (Supplementary Fig. 1), which may reflect an attempt to establish social dominance among unfamiliar mice [31].

To understand the neural basis for reduced writhing behavior in stranger dyads when compared with cagemate dyads, a subset of mice from each social condition was selected for brain region-specific mapping of c-Fos expression. We considered several brain areas known to participate in pain modulation, social cognition, and threat, including the mPFC, ACC, and amygdala [6, 32]. There was a significant main effect of social context (alone vs. cagemate vs. stranger) for the prelimbic subdivision of the mPFC (Fig. 1d–f) and the ACC (Fig. 1d–f), with no other brain region reaching statistical significance (All Fs <2.086, all ps > 0.144; Supplementary Fig. 2). Fos labeling was significantly increased in stranger dyads for both brain regions compared to mice tested alone and cagemate dyads, suggesting enhanced neural activity within these brain regions (Fig. 1f). In the stranger condition, there was a significant negative correlation between writhing behavior and c-Fos labeling for both the prelimbic (Fig. 1g) and the ACC (Fig. 1h). There was also a significant positive correlation between writhing behavior and c-Fos labeling in the paraventricular nucleus (PVN) of the hypothalamus (Fig. 1i). However, cell-type-specific activation of these regions was not measured; therefore, it remains unknown whether certain cell-types or receptor populations are involved in inhibiting pain behaviors in strangers.

### Glucocorticoid receptor activity is enhanced in the prelimbic cortex of stranger dyads

**Fig. 2:**
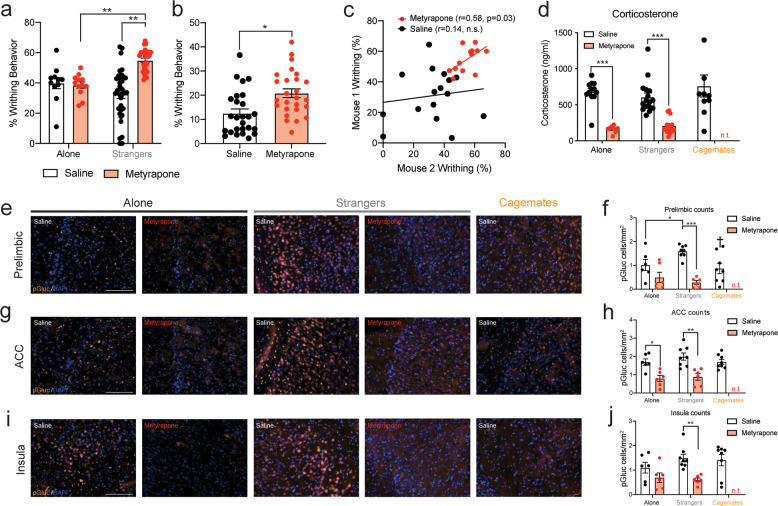
Inhibition of the HPA-stress axis induces pain contagion in stranger mice and reduces glucocorticoid activity in the prelimbic cortex. **a** Metyrapone (50 mg/kg), enhances writhing behavior in mice tested in stranger dyads (*n* = 26), when compared with vehicle-treated dyads (*n* = 32) and mice tested alone, both vehicle-treated (*n* = 11) and metyrapone-treated (*n* = 12) (two-way ANOVA, main effect of drug: *F*_1,77_ = 1.76, *p* > 0.05; main effect of social context: *F*_1,77_ = 12.28, *p* < 0.001; social context × drug interaction: *F*_1,77_ = 15.41, *p* < 0.001). **b** Higher co-occurrence of writhing behavior in metyrapone-treated compared with vehicle-treated dyads. Using data from **a**, the expected number of samples with writhing in both mice of the dyad was calculated as a joint probability. Bars represent the mean ± SEM of episodes of joint writhing expressed as a percentage (independent samples *t*-test, *t*_50_ = 3.25, *p* < 0.01). **c** Writhing behavior is significantly correlated between mice within metyrapone-treated **(***r* = 0.58, *p* = 0.03), but not vehicle-treated dyads (*r* = 0.14, n.s). **d** Blood plasma corticosterone is similar between mice tested alone (*n* = 12), with a stranger (*n* = 16) or with a cagemate *(n* = 10) (one-way ANOVA, *F*_2,35_ = 0.79, *p* > 0.05). However, metyrapone significantly reduced blood plasma corticosterone in mice tested alone (*n* = 9) and stranger dyads (*n* = 16) (two-way ANOVA, main effect of drug: *F*_1,51_ = 102.2, *p* < 0.001; main effect of social: *F*_1,51_ = 0.08, *p* > 0.05; social context × dug interaction: *F*_1,51_ = 1.72, *p* > 0.05). **e** Representative images showing phosphorylated glucocorticoid receptor (p-GR) staining in the prelimbic cortex for mice tested alone or within a saline-treated or metyrapone-treated stranger dyad. A representative image for the cagemate condition is also shown. **f** p-GR (p-GR cells/mm^2^) staining in the prelimbic cortex is higher in stranger (*n* = 8) compared with cagemate dyads (*n* = 8) (one-way ANOVA, *F*_2,19_ = 4.339, †*p* < 0.05 compared with stranger dyads). p-GR staining in the prelimbic is significantly reduced by pre-treatment with metyrapone in stranger dyads, but not mice tested alone (two-way ANOVA, main effect of drug: *F*_1,22_ = 31.06, *p* < 0.001; main effect of social: *F*_1,22_ = 1.16, *p* > 0.05; social context × drug interaction: *F*_1,22_ = 5.72, *p* < 0.01). **g** Representative images showing p-GR staining in the anterior cingulate cortex (ACC) for mice tested alone or within a saline- or metyrapone-treated stranger dyad. A representative image for the cagemate condition is also shown. **h** p-GR staining in the ACC is similar between mice tested alone or within a stranger or cagemate dyad (one-way ANOVA, *F*_2,19_ = 1.02, *p* > 0.05). Metyrapone significantly reduced p-GR staining in the ACC of mice tested alone (*n* = 6) or with a stranger (*n* = 8) (two-way ANOVA, main effect of drug: *F*_1,22_ = 29.20, *p* < 0.001; main effect of social context: *F*_1,22_ = 1.06, *p* > 0.05; social context × drug interaction: *F*_1,22_ = 0.32, *p* > 0.05). **i** Representative images showing p-GR staining in the insular cortex for mice tested alone or within a saline-treated or metyrapone-treated stranger dyad. A representative image for the cagemate condition is shown for comparison. **j** p-GR staining in the insula is similar between mice tested alone or within a stranger or cagemate dyad (one-way ANOVA, *F*_2,19_ = 0.91, *p* > 0.05). Metyrapone significantly reduced p-GR staining in the insula of mice tested alone and within a stranger dyad (two-way ANOVA, main effect of drug: *F*_1,22_ = 14.18, *p* < 0.001; main effect of social context: *F*_1,22_ = *0.75, p* > 0.05; social context × drug interaction: *F*_1,22_ = 1.99, *p* > 0.05). For two-way ANOVA cagemates were not included in the analyses because the experimental design did not include a metyrapone-treated cagemate group, a decision made based on previous work [11]. Scale bars = 100 μM. *p < 0.05, **p < 0.01, ***p < 0.001.

Since we previously showed that stress blocks pain contagion in strangers [11], we next sought to identify whether social interactions with a stranger mouse were sufficient to enhance glucocorticoid receptor activity in specific brain regions, and determine whether blocking glucocorticoid synthesis reversed these changes. Mice were pre-treated with the glucocorticoid synthesis inhibitor metyrapone (50 mg/kg, s.c.), at a dose that enhances co-writhing in stranger dyads [11, 33]. As shown in Fig. 2a, metyrapone enhanced writhing in stranger dyads compared with vehicle-treated dyads, replicating our previous work [11]. Metyrapone also enhanced the co-occurrence of writhing behavior (Fig. 2b), and metyrapone-treated dyads exhibited highly correlated writhing behavior (Fig. 2c). Corticosterone levels were not higher in stranger dyads than in cagemate dyads or mice tested alone (Fig. 2d), but metyrapone reduced blood corticosterone levels in both the alone and stranger condition (Fig. 2d). To assess whether metyrapone specifically reduced GR activity in the brain, we measured GR phosphorylation (p-GR) at Ser(211), a known biomarker for GR activation in vivo [34] in regions identified by our c-Fos analysis (i.e., prelimbic cortex, ACC) and the insula, a region important for vicarious pain responses in humans [35]. Of the regions examined, only the prelimbic cortex of stranger mice exhibited more p-GR staining than the alone condition and cagemate dyads, which was abolished by pre-treatment with metyrapone (Fig. 2e, f). The presence of a stranger mouse did not enhance p-GR in the ACC (Fig. 2g, h) or insula (Fig. 2g, h), but metyrapone reduced p-GR in these regions in stranger dyads (Fig. 2i, j). Immunoblotting supported these data, where stranger dyads showed increased p-GR in the mPFC (but not other regions) (Supplementary Fig. 3), which was significantly reduced by metyrapone pre-treatment (Supplementary Fig. 4) and negatively correlated with corticosterone in saline-treated stranger dyads (*r* = −0.588, *p* < 0.05; Supplementary Fig. 5). In the absence of a nociceptive stimulus, blood corticosterone and p-GR in the prelimbic cortex were not increased in stranger dyads indicating that social context and nociceptive processes interact to enhance GR activity, and potentially modulate writhing behavior within a social context (Supplementary Fig. 6).

### Targeted inhibition of glucocorticoid receptors in the prelimbic cortex reveals pain contagion in stranger dyads

**Fig. 3:**
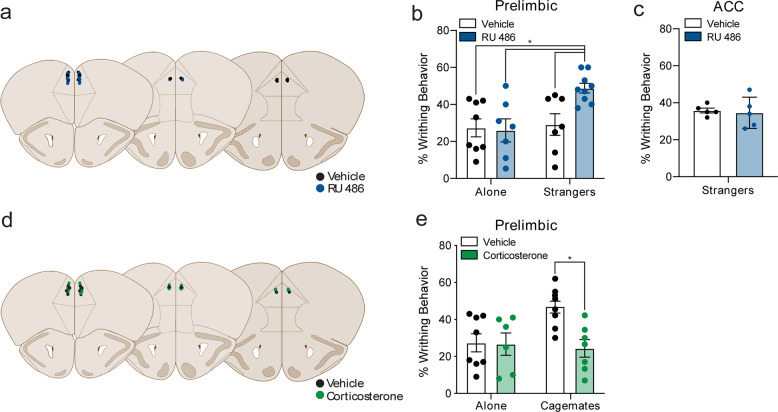
Selectively targeting glucocorticoid receptors in the prelimbic cortex modulates pain contagion. **a** Schematic illustration showing the center of the microinjection sites within the prelimbic cortex for vehicle and RU 486. **b** Bilateral microinjections of the glucocorticoid receptor antagonist RU 486 in the prelimbic cortex enhance writhing behavior in mice tested in stranger dyads (*n* = 9) when compared with vehicle-injected stranger dyads (*n* = 7), and mice tested alone, both vehicle-injected (*n* = 8) and RU 486-injected (*n* = 7) (two-way ANOVA, main effect of social context: *F*_1,27_ = 6.37, *p* = 0.02; social context × drug interaction: *F*_1,27_ = 4.67, *p* < 0.05). **c** Bilateral microinjections of RU 486 within the anterior cingulate cortex do not enhance writhing behavior in stranger dyads (*n* = 5) when compared with vehicle-injected stranger dyads (*n* = 5) (*t*_8_ = 0.3, *p* = 0.77). **d** Schematic illustration showing the center of the microinjection sites within the prelimbic cortex for vehicle and corticosterone. **e** Bilateral microinjections of the glucocorticoid receptor agonist corticosterone in the prelimbic cortex, decreased writhing behavior in mice tested in cagemate dyads *(n* = 7) when compared with vehicle-injected cagemate dyads (*n* = 8) and mice tested alone, both vehicle-injected (*n* = 8, replotted from **b**) and corticosterone-injected (*n* = 6) (two-way ANOVA, main effect of drug: *F*_1,24_ = 4.96, *p* < 0.05; social context × drug interaction: *F*_1,24_ = 4.3, *p* < 0.05). *p < 0.05.

To test whether GR activity specifically within the prelimbic cortex of stranger mice modulates pain expression within a social context, we bilaterally microinjected RU 486, a GR antagonist, into the prelimbic cortex of stranger dyads. Figure 3a illustrates the microinjection sites for each surgery, color-coded depending on whether vehicle or RU 486 was administered. Strikingly, inhibition of GRs in the prelimbic cortex of stranger dyads enhanced pain contagion but did not change writhing behavior in mice tested alone (Fig. 3b). In contrast, RU 486 microinjection into the ACC did not enhance pain contagion in strangers relative to vehicle-injected controls (Fig. 3c). In some mice (*n* = 3), cannula placement was located in the infralimbic cortex, where RU 486 did not enhance writhing behavior in stranger dyads (Supplementary Fig 7). Finally, to show that increased GR activity within the prelimbic cortex suppressed pain contagion, we activated GRs in the prelimbic cortex of cagemate dyads with bilateral microinfusions of corticosterone (Fig. 3d). As shown in Fig. 3e, prelimbic microinfusions of corticosterone blocked pain contagion in cagemate dyads. Representative images for the spread of a fluorescently labeled ligand microinjected into the prelimbic cortex are shown in Supplementary Fig 8. Together, these results suggest that GR activity in the prelimbic cortex bidirectionally modulates pain contagion within a social context.

### Increased synaptic drive of prelimbic neurons in stranger dyads and reversal by metyrapone pre-treatment

**Fig. 4:**
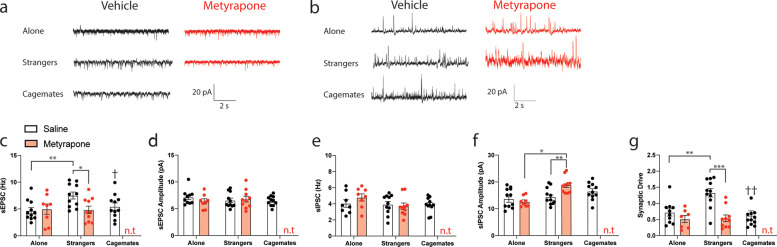
Synaptic transmission is enhanced in the prelimbic cortex of stranger mice. **a** Representative prelimbic layer II/III spontaneous excitatory postsynaptic current (sEPSC) traces recorded following behavioral testing for mice tested alone or with a stranger and either treated with saline (black) or metyrapone (red). A representative trace for the cagemate condition is also shown. **b** Representative prelimbic layer II/III spontaneous inhibitory postsynaptic current (sEPSC) traces recorded following behavioral testing for mice tested alone or with a stranger and either treated with saline (black) or metyrapone (red). A representative trace for the cagemate condition is also shown. **c** sEPSC frequency (Hz) is enhanced in stranger dyads compared with mice tested alone and cagemate dyads (one-way ANOVA, *F*_2,30_ = 6.42, ^†^*p* < 0.05, ^††^*p* < 0.01 compared with stranger dyads, all vehicle-treated conditions). sEPSC frequency (Hz) is normalized by metyrapone pre-treatment in stranger dyads (two-way ANOVA, main effect of drug: *F*_1,36_ = 3.43, *p* = 0.07; main effect of social context: *F*_1,36_ = 4.25, *p* < 0.05; social context × drug interaction: *F*_1,36_ = 5.16, *p* < 0.05). **d** sEPSC amplitude (pA) is not significantly different between vehicle-treated mice tested alone or within a stranger or cagemate dyad (one-way ANOVA, *F*_2,30_ = 1.90, *p* > 0.05). Metyrapone pre-treatment did not alter sEPSC amplitude (pA) in mice tested alone or within a stranger dyad (two-way ANOVA, main effect of drug: *F*_1,36_ = 1.14, *p* > 0.05; main effect of social context: *F*_1,36_ = 0.65, *p* > 0.05; social context x drug interaction: *F*_1,36_ = 1.90, *p* > 0.05). **e** sIPSC frequency (Hz) is not significantly different between vehicle-treated mice tested alone or within a stranger or cagemate dyad (one-way ANOVA, *F*_2,30_ = 0.05, *p* > 0.05). Metyrapone pre-treatment did not alter responses in mice tested alone or within a stranger dyad (two-way ANOVA, main effect of drug: *F*_1,36_ = 0.40, *p* > 0.05; main effect of social context: *F*_1,36_ = 2.12, *p* > 0.05; social context × drug interaction: *F*_1,36_ = 1.36, *p* > 0.05). **f** sIPSC amplitude (pA) is not significantly different between vehicle-treated mice tested alone or within a stranger or cagemate dyad (one-way ANOVA, *F*_2,30_ = 1.39, *p* > 0.05). Metyrapone pre-treatment enhanced sIPSC amplitude (pA) compared with mice tested alone and vehicle-treated stranger dyads (two-way ANOVA, main effect of drug: *F*_1,36_ = *1.83, p* > 0.05; main effect of social context: *F*_1,36_ = 10.57, *p* < 0.01; social context × drug interaction: *F*_1,36_ = 9.44, *p* < 0.01). **g** Synaptic drive is increased in stranger dyads compared with mice tested alone or within a cagemate dyad (one-way ANOVA, *F*_2,30_ = 8.80, ^†††^*p* < 0.01 compared with stranger dyads, all vehicle-treated conditions). Synaptic drive is normalized by metyrapone pre-treatment in stranger dyads (two-way ANOVA, main effect of drug: *F*_1,36_ = 17.15, *p* < 0.001; main effect of social context: *F*_1,36_ = 6.97, *p* = 0.012; social context × drug interaction: *F*_1,36_ = 6.01, *p* = 0.019). Similar to Fig. 2, a metyrapone-treated cagemate condition was not included based on previous work [11]. *n* = 9–11 cells/group prepared from 4 to 5 mice. *p < 0.05, **p < 0.01, ***p < 0.001.

We next sought to determine whether neuronal function was altered in the prelimbic cortex of mice following social interactions with another mouse in pain and assess whether pre-treatment with metyrapone normalized this activity. Thus, we used slice electrophysiology and recorded spontaneous synaptic transmission in layer II/III of the prelimbic cortex immediately following behavioral testing. For each condition, four mice were used with sample sizes ranging between 9 and 11 cells/group. Representative traces for sEPSCs (Fig. 4a) and sIPSCs (Fig. 4b) in the prelimbic cortex after behavioral testing are shown for each of the social conditions with or without metyrapone (50 mg/kg, s.c.) pre-treatment. The frequency, but not the amplitude of sEPSCs, was significantly increased in stranger dyads when compared with mice tested alone (Fig. 4b, c), and metyrapone pre-treatment reversed this increase (Fig. 4b, c). There was no effect for social context or metyrapone pre-treatment on sIPSC frequency (Fig. 4e); however, sIPSC amplitude was increased by metyrapone treatment but only in stranger dyads (Fig. 4f). The increased sIPSC amplitude in metyrapone-treated strangers was comparable to that of cagemate dyads (*t*_19_ = 1.78, *p* > 0.05). To better understand the impact of social context and metyrapone pre-treatment on synaptic transmission, we calculated the synaptic drive, an overall measure of synaptic functioning for each neuron. Consequently, synaptic drive was enhanced in stranger dyads, and this was reversed by metyrapone-treatment (Fig. 4g).

### Social interactions affect the expression of pain differently in cagemate and stranger mice

**Fig. 5:**
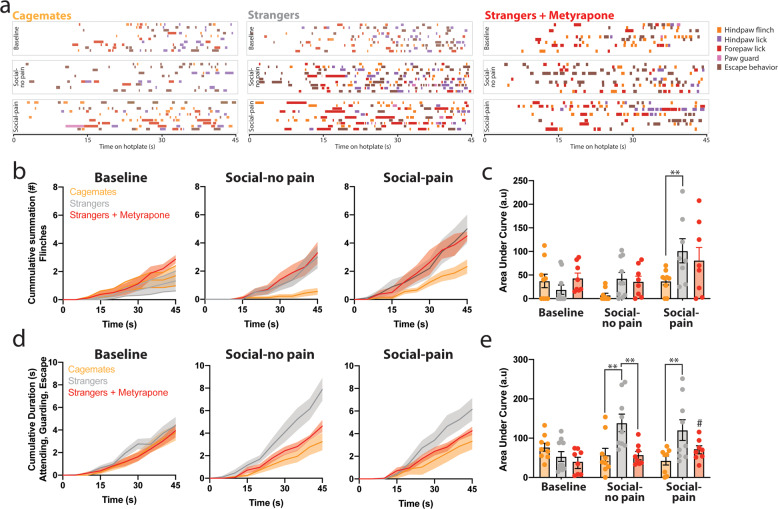
Enhanced nociceptive and affective-motivational pain behavior in stranger mice following social interactions. **a** Temporal raster plot for behaviors exhibited during placement in an inescapable noxious environment (enclosed 52.5 °C hotplate). Nociception-induced sensory-reflexive (flinching) and affective-motivational (paw licking, guarding, and escape) behaviors were measured during three phases of the experiment: before any social interaction (baseline), following a 30 min social interaction with either a cagemate or stranger (social-no pain), and following acetic acid injection while in the cagemate or stranger dyad (social-pain). A separate group of stranger dyads was injected with metyrapone before baseline testing. Each row displays the measured behaviors over 45 s for an individual mouse (cagemates: *n* = 10; strangers: *n* = 10, strangers + metyrapone: *n* = 8). **b** Cumulative summation of paw flinches in panel **a** for baseline, social-no pain, and social-pain phases of the experiment for cagemates, strangers, and strangers-treated with metyrapone. **c** Area under the curve (AUC) analysis for nociceptive behaviors for baseline, social-no pain and social-pain phases of the experiment (two way repeated measures (RM) ANOVA, main effect of social context: *F*_2,25_ = 3.40, *p* = 0.04; main effect of phase (RM): *F*_2,50_ = 7.92, *p* < 0.0001; social context × phase of testing: *F*_4,50_ = 2.08, *p* = 0.09) **d** Cumulative summation of affective-motivational behaviors in **a** for baseline, social-no pain, and social-pain phases of the experiment for cagemates, strangers and strangers-treated with metyrapone. **e** Area under the curve (AUC) analysis for affective-motivational behaviors for baseline, social-no pain, and social-pain phases of the experiment (two way repeated measures ANOVA, main effect of social context: *F*_2,25_ = 8.82, *p* < 0.0001; main effect of phase (RM): *F*_2,50_ = 2.562, *p* = 0.08; social context × phase of testing: *F*_4,50_ = 4.063, *p* < 0.01). Post-hoc com*p*arisons were only conducted within each testing phase (baseline, social-no pain, and social-pain) by Tukey’s post hoc test. **p < 0.01, #*p* = 0.07 compared with analogous stranger condition.

In stranger mice, we reasoned that enhanced GR activity and synaptic functioning in the prelimbic cortex might reflect the detection of a social threat, which adaptively may work to suppress overt pain behavior in the presence of an unfamiliar conspecific. Since social threat leads to decreased pain expression, but a simultaneous increase in reported pain intensity [15, 16], we assessed whether pain behavior was increased in strangers following removal from dyadic conditions. Thus, mice were individually placed on an inescapable thermal stimulus for 45 s [23] and assessed for nociceptive reflexes and affective-motivational responses at three timepoints during the testing session (baseline, following a social interaction (social-no pain) and acetic acid testing (i.e., social-pain) (Fig. 5a–e). The cumulative summation of nociceptive responses and cumulative duration of affective-motivational behavior was similar between mice in the cagemate and stranger groups during the 45 s baseline assessment (nociceptive: *t*_17_ = 1.09, *p* = 0.29; affective-motivational behavior: *t*_17_ = 1.44, *p* = 0.17, Fig. 5a–e). Nociceptive reflexes were increased in stranger dyads following social-pain; however, metyrapone pre-treatment did not change nociceptive responding in stranger dyads (Fig. 5a–c). Also, affective-motivational behaviors were increased in stranger dyads following social-no pain and social-pain, which were prevented with metyrapone treatment (Fig. 5a, d, e). In addition, we examined the latency to first response (i.e., the most commonly used hotplate metric) for either nociceptive (i.e., flinch) or affective-motivational behavior (i.e., hind paw lick). The latency to hindpaw flinch was not significantly different between the conditions at any phase of testing (Supplementary Fig. 9a); however, the latency to hindpaw lick was lower in stranger dyads following social-no pain and social-pain with metyrapone treatment reversing this trend (Supplementary Fig. 9b).

## Discussion

The importance of brain regions such as the medial prefrontal cortex [10], anterior cingulate cortex [4, 5], and insular cortex [36] are not only critical for rodent empathy but also human empathy [37]. This suggests conserved neuronal mechanisms are likely to be found between the species. The experiments described here identify the prelimbic subdivision of the medial prefrontal cortex as an important site for bidirectionally modulating pain contagion in mice. Glucocorticoid receptor activity within the prelimbic cortex modulated writhing behavior when a social partner was present but did not when mice were tested alone. We further showed using electrophysiology, that following a social interaction with a stranger in pain, there was increased excitatory synaptic transmission in the prelimbic cortex of mice while pre-treatment with metyrapone reversed these changes. Removing stranger mice from dyadic testing revealed latent pain behavior when placed singly on an inescapable thermal surface, which was reduced by metyrapone.

Socially affective behaviors like pain contagion [7, 11], fear learning [5, 38], social approach [39, 40], and stress contagion [41]—especially in mice—are largely dependent on familiarity. In particular, pain contagion is determined by the nature of the social relationship [7], type of social interaction [14, 42], and pain status of those interacting [39]. We previously reported that social stress prevented pain contagion in stranger dyads through greater HPA activity, but these observations were based on behavioral pharmacology [11]. The current data now show that plasma corticosterone levels were not higher in stranger dyads than in cagemate dyads or mice tested alone, but rather strangers have a different pattern of GR activity in prefrontal cortical regions. This activity is further highlighted by increased excitatory synaptic transmission in the prelimbic cortex of stranger mice following dyadic pain testing and the subsequent reversal of this effect with metyrapone pre-treatment. As different stressors result in unique levels of catecholamines (noradrenaline and adrenaline) and corticosterone release, GR activity patterns may differ based on the type of stressor (social vs. non-social). While corticosterone is just one physiological marker of stress reactivity, it is an important endpoint of the HPA axis and an index of limbic activity [43]. Social stress would expectedly increase limbic activity at the level of stress-appraisal or social threat [44], which would involve brain regions important for social cognition that would, in turn, trigger the hypothalamus [45]. Further, our results align with previous observations showing that behavioral stressors cause long-lasting changes in glutamatergic synaptic transmission in the mPFC [29, 46].

Analysis of c-Fos immunoreactivity across several limbic ROIs identified only two regions (prelimbic cortex and ACC), where c-Fos expression was elevated in the stranger, but not cagemate dyads. Activity within these regions typically serves as important areas for acute and chronic pain modulation [47, 48] and social threat detection [6, 29, 49]. While c-Fos expression was increased in the prelimbic and ACC of stranger dyads, only inhibition of GRs within the prelimbic cortex revealed pain contagion. Microinjections of RU-486 into the prelimbic, but not the ACC revealed pain contagion in stranger dyads, while corticosterone microinjections into the prelimbic cortex blocked pain contagion in cagemate dyads. Given the proximity of the prelimbic and ACC (approximately 350 μm) and highly lipophilic properties of RU 486 and corticosterone (i.e., ability to diffuse from prelimbic to ACC), it could be possible for the ligands to traverse cytoarchitectural boundaries. To provide confidence that the ligands did not cross the prelimbic and ACC boundary, we microinjected a fluorescent ligand (muscimol, another highly lipophilic substance) into the prelimbic allowing us to visualize injection spread, which was mostly contained to the prelimbic cortex.

We suspect that the prelimbic cortex may operate as a gate to either allow or suppress the expression of nociceptive behavior in the presence of a social partner. In humans, the dorsal medial PFC—analogous to the rodent prelimbic cortex—is consistently activated by social behaviors requiring the perception of dissimilar others [49]. Another possibility is that centrally released glucocorticoids blocked pain contagion in stranger dyads through selective activation of the prelimbic cortex. For instance, corticotrophin-releasing factor (CRF) is known to play a role in anxiogenic responses through a mPFC mechanism [50], and exposure to predator scent leads to CRF receptor 1-dependent changes in synaptic function, which are specific to the prelimbic cortex [29]. However, the central release of glucocorticoids is not a likely mechanism because systemic metyrapone reduced p-GR in the prelimbic and normalized synaptic drive in stranger dyads. GRs within the prelimbic cortex act as a major site for feedback of HPA responses because local glucocorticoid infusions inhibit anticipatory (but not reflexive) responses to stressors [51]. Activation of GRs within the prelimbic cortex of stranger dyads—when both mice were injected with acetic acid—may suggest enhanced negative feedback to the HPA axis in strangers, which would counteract any physiological corticosterone increase in strangers. However, in the absence of a nociceptive stimulus, p-GR was similar for all social conditions suggesting that pain contagion was inhibited through a specific feedback mechanism. The anticipatory nature of the unfamiliar social interaction may increase the neural control of innate defense programs that would suppress pain expression in the presence of a social threat [52].

Since displaying pain is a liability for many animals because it signals weakness [53], we explored whether stranger dyads may be suppressing overt pain expression while exhibiting sensitivity to other pain modalities. The influence of social threat on overt pain behavior (i.e., acetic acid writhing) was separated from nociceptive and affective-motivational pain responses by momentarily removing mice from the dyad and testing them on an inescapable thermal apparatus. In strangers, nociceptive behavior in response to noxious heat on the hotplate was increased immediately after the acetic acid test, while affective behaviors were increased following social interactions and immediately after acetic acid testing. Increased nocifensive behavior on the hotplate following the acetic acid test may be due to a latent effect of acetic acid on the mouse’s nociceptive responses as we have shown that 0.6% acetic acid—the concentration used in the current study—increased thermal pain sensitivity [54]. However, we would like to point out that all mice in the current study received acetic acid before hotplate testing and observed differences were only noted within stranger dyads. Further, metyrapone pre-treatment reversed affective-motivational behaviors in strangers following social interactions but did not affect nociceptive responses. Since metyrapone also reversed increased synaptic drive in the prelimbic and targeted inhibition of GRs in the prelimbic revealed pain contagion in strangers, we believe that our data collectively support the notion that GR activity in the prelimbic cortex modulates social interaction-induced changes in nociceptive behavior.

In sum, we find that GRs in the prelimbic cortex are critical for modulating pain responses within a social context. Stranger mice do not display pain contagion but following removal from the social context show enhanced nociceptive reflexes. This enhanced nociceptive processing is consistent with pain processing during social threat in humans [16]. The inhibition or activation of GRs in the prelimbic cortex enhanced or inhibited pain contagion in stranger and cagemate dyads, respectively. We also identified enhanced excitatory synaptic transmission in the prelimbic cortex of stranger dyads. Together this may indicate that increased glucocorticoid input increases synaptic drive in the prelimbic cortex of strangers to reduce pain when in the presence of a social partner but may enhance latent nociceptive sensitivity once removed from the dyad. In the future, it will be essential to determine whether there are specific downstream targets for activated cells within the prelimbic cortex, that modulate pain contagion and compare specific cellular populations that become activated during the observation of a familiar or unfamiliar conspecific in pain.

## Funding and disclosure

This research was supported by the Ontario Ministry of Innovation (Early Career Researcher Award to LJM), Natural Sciences and Engineering Research Council of Canada (NMF, LJM), Canadian Foundation for Innovation (NMF, LJM), and the Canada Research Chairs program (LJM). All authors have nothing to disclose.

## Supplementary information

Supplemental material
