# Supplementary material for: Prelimbic cortex glucocorticoid receptors regulate the stress-mediated inhibition of pain contagion in male mice
Source: Neuropsychopharmacology. 2020 Nov 23;46(6):1183–93. doi: 10.1038/s41386-020-00912-4 (PMC8115346; doi:10.1038/s41386-020-00912-4)
Supplement: Supplementary file 1 — Supplemental material [file 41386_2020_912_MOESM1_ESM.docx]

**Supplementary Methods**

**Western blot**

Immediately following behavior, a subset of mice was randomly selected, and their brain quickly removed and microdissected. The brain was placed in an adult mouse brain matrix (Kent Scientific, CT) and coronal sections (500 μm) were prepared. Using a Leica binocular M60 stereoscope, the regions corresponding to the mPFC, ACC, BNST, and hypothalamic PVN were visually identified and microdissected. Tissue extracts were prepared in ice-cold homogenization buffer containing (in mM): 50 Tris-HCl, pH 7.4; 150 NaCl; 1 EDTA; 1% Triton X-100; 5 NaF; 1.5 Na3VO4 and protease inhibitor cocktail (complete, EDTA-free Roche Applied Science, Indianapolis, IN). Following centrifugation at 12,000 × g for 10 min at 4 °C, the supernatant protein concentration was measured, and equal protein quantities were boiled for 5 min in sample buffer and separated by SDS-PAGE. Following electrophoresis, proteins were transferred to 0.2 mm nitrocellulose membranes. Membranes were blocked in 5% dry milk powder in Tris-buffered saline containing 0.1% Tween-20 (TBS-T) for 1 h before overnight incubation with primary antibody. The membranes were then washed, incubated for 1 h with HRP-conjugated secondary antibody, washed again, treated with Enhanced Chemiluminescence reagent (Perkin Elmer) followed by chemiluminescence detection with the ImageQuant LAS 500 imager (GE Healthcare Life Sciences, Mississauga, ON). All signals were obtained in the linear range for each antibody, and densitometric analyses were performed with Image J (National Institutes of Health, Bethesda, MD. Protein expression levels were expressed as the ratio of phosphoprotein to total protein (with each band normalized to β-actin). This value was then used to make comparisons between social conditions using one-way ANOVA. The antibodies and dilutions for the Western blots used in these studies are as follows: phospho-glucocorticoid receptor (Ser211) (1:1000, Cat. #4161, Cell Signaling Technology), glucocorticoid Receptor (D6H2L) (1:1000, Cat. #12041, Cell Signaling Technology), β‑actin (1:5000, Cat. #12620, Cell Signaling Technology).


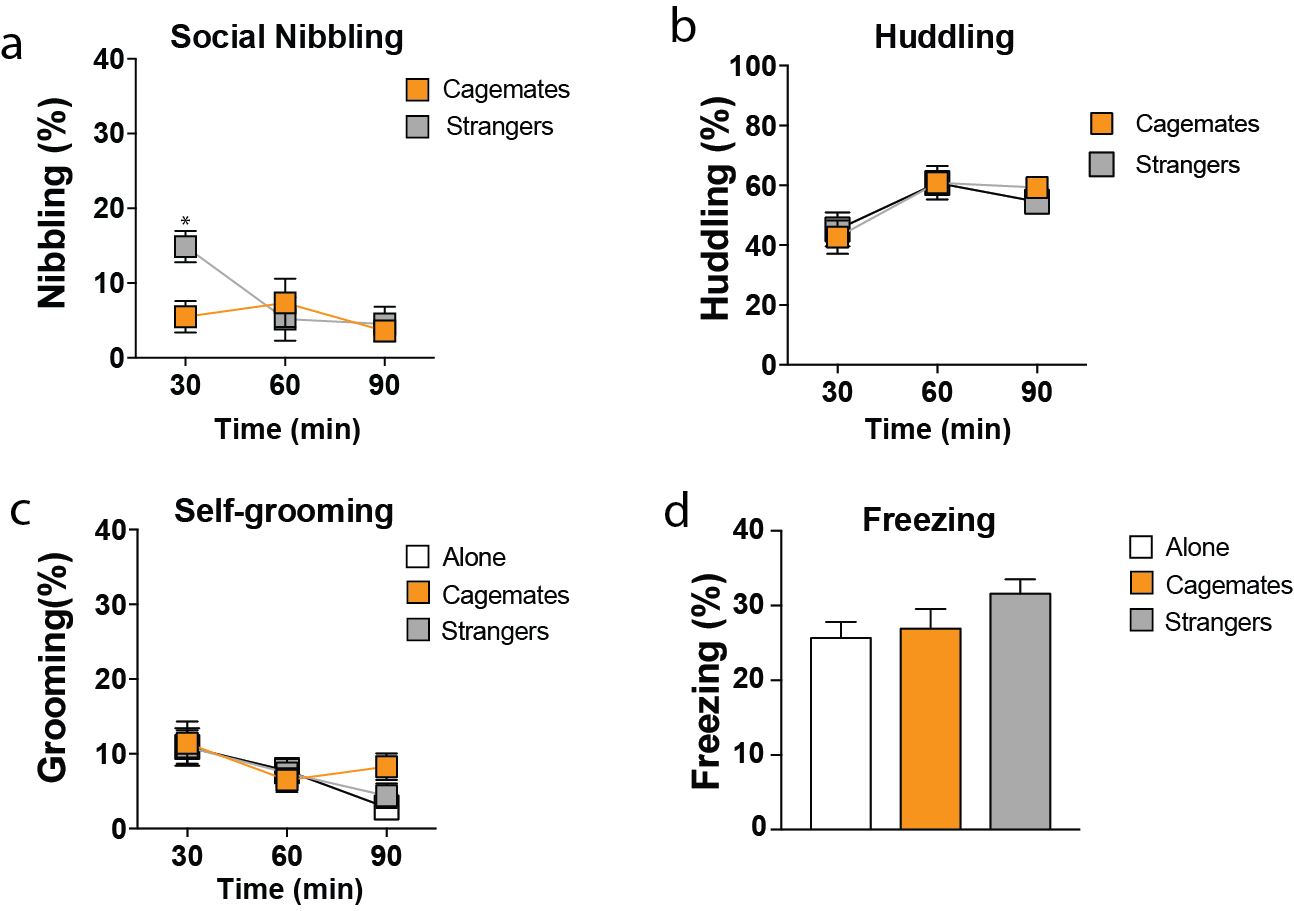


**Supplemental Figure 1.** Behavior displayed by mice following acetic acid injection. **a.** Social nibbling was higher among stranger dyads during the first 30 min following acetic acid injection when compared with cagemates (two-way repeated measures ANOVA, *main effect of social context:* *F_1,22_*=1.93, *p*=0.17; *main effect of time (RM):* *F_2,44_*=3.38, *p*=0.04; *social context x time of testing:* *F_2,44_*=3.14, *p*=0.04). **b.** Social huddling, defined as bodily touching was not significantly different between cagemates and stranger dyads (two-way repeated measures ANOVA, *main effect of social context:* *F_1,22_*=0.02, *p*=0.87; *main effect of time (RM):* *F_2,44_*=36.95, *p*<0.0001; *social context x time of testing:* *F_2,44_*=0.96, *p*=0.39). **c** Self-grooming behavior was not significantly different between the different social conditions (two-way repeated measures ANOVA, main effect of social context: *F_2,31_*=0.48, *p*>0.05; main effect of time (RM): *F_2,62_*=6.74, *p*<0.05; social context x RM: *F_4,62_*=0.81, *p*>0.05). **d** Freezing over 90 min was not significantly different between the different social conditions (one-way ANOVA, *F_2,31_*=1.89, *p*=0.165). Bars represent mean ± S.E.M. Sample sizes were n=10 for mice tested alone, n=12 for cagemates and n=12 for strangers. **p*<0.05.


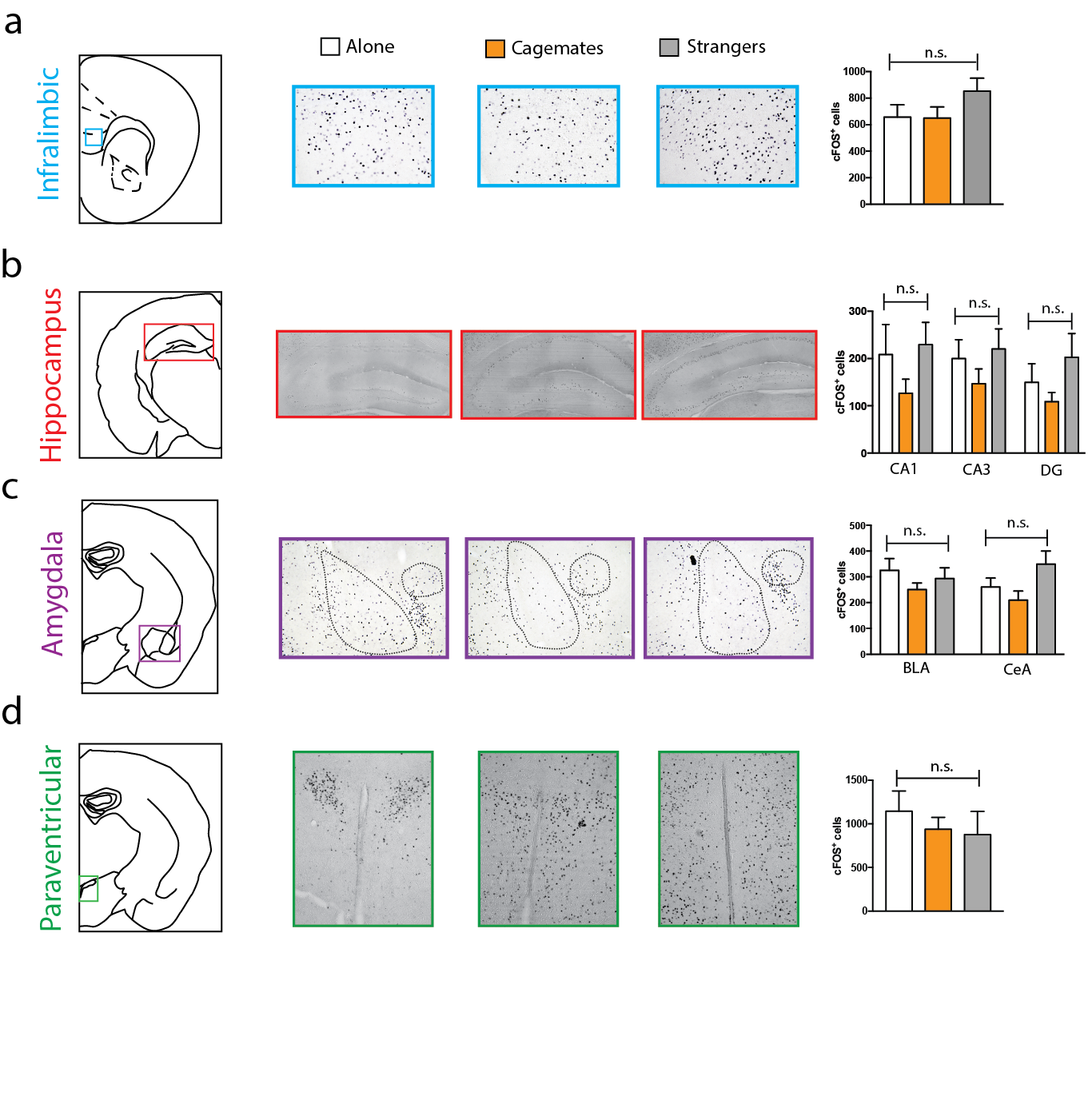


**Supplementary Figure 2.** c-FOS expression is not different between the social conditions in the **a** infralimbic subdivision of the medial prefrontal cortex (mean ± S.E.M., one-way ANOVA, *F_2,31_*=1.62, p>0.05), **b** hippocampus subregions (mean ± S.E.M., one-way ANOVA, *CA1:* *F_2,31_*=1.23, *p*>0.05; *CA3*: *F_2,31_*=0.96, *p*>0.05; *DG:* *F_2,31_*=1.44, *p*>0.05), **c** amygdala (mean ± S.E.M., one-way ANOVA, *BLA:* *F_2,31_*=0.92, *p*>0.05; *CeA:* *F_2,31_*=0.73, *p*>0.05*),* and **d** paraventricular nucleus of the hypothalamus (mean ± S.E.M., one-way ANOVA, *F_2,31_*=0.40, *p*>0.05). Illustrations showing the target areas analyzed are presented in each panel along with representative images showing c-Fos staining for each condition. Sample sizes were n=10 for mice tested alone, n=11 for cagemates and n=12 for strangers. DG = dentate gyrus, BLA = basal lateral amygdala, CeA = central amygdala. n.s. = not significant.


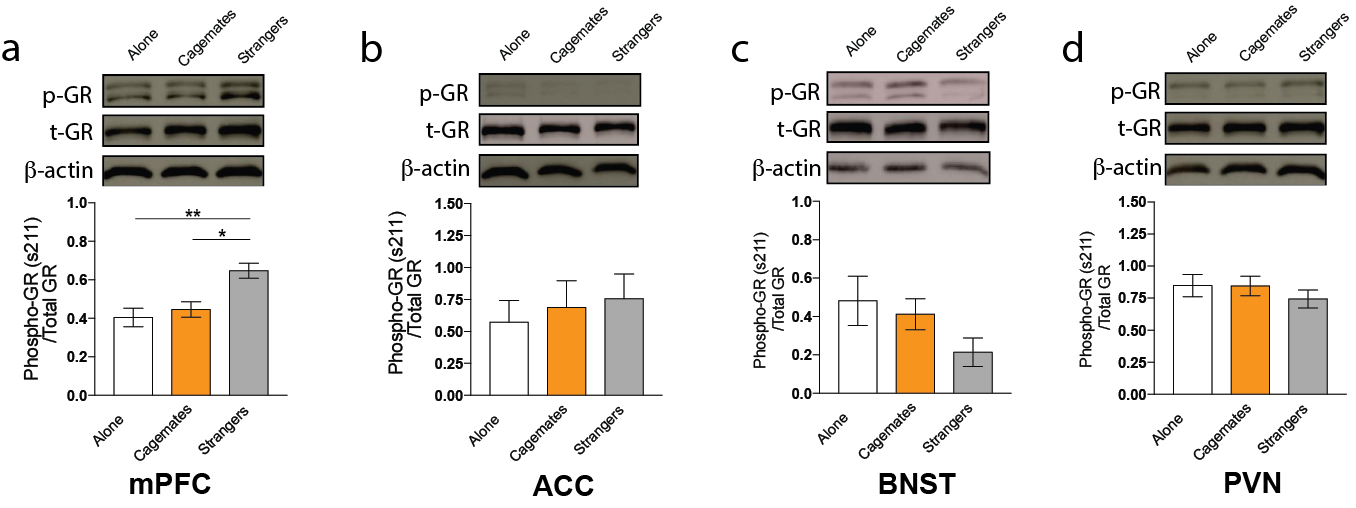


**Supplementary Figure 3.** Western blot analysis showing phosphorylated glucocorticoid (p-GR) receptor expression in brain regions associated with contagion and stress. **a** Increased p-GR expression in the medial prefrontal of stranger dyads following co-writhing behavior (one-way ANOVA, F*_2,15_*=8.72, p=0.003). **b** No difference in p-GR expression in the anterior cingulate cortex (ACC) between the social conditions (one-way ANOVA, F*_2,15_*=0.2, *p*>0.05). **c** No difference in p-GR expression in the bed nucleus of the stria terminalis (BNST) between the social conditions (one-way ANOVA, F*_2,15_*=1.8, *p*>0.05). **d** No difference in p-GR expression in the paraventricular nucleus of the hypothalamus between the social conditions (one-way ANOVA, F*_2,15_*=1.6, *p*>0.05). *n*=6/condition, bars represent mean ± S.E.M. * p<0.05; **p<0.001.


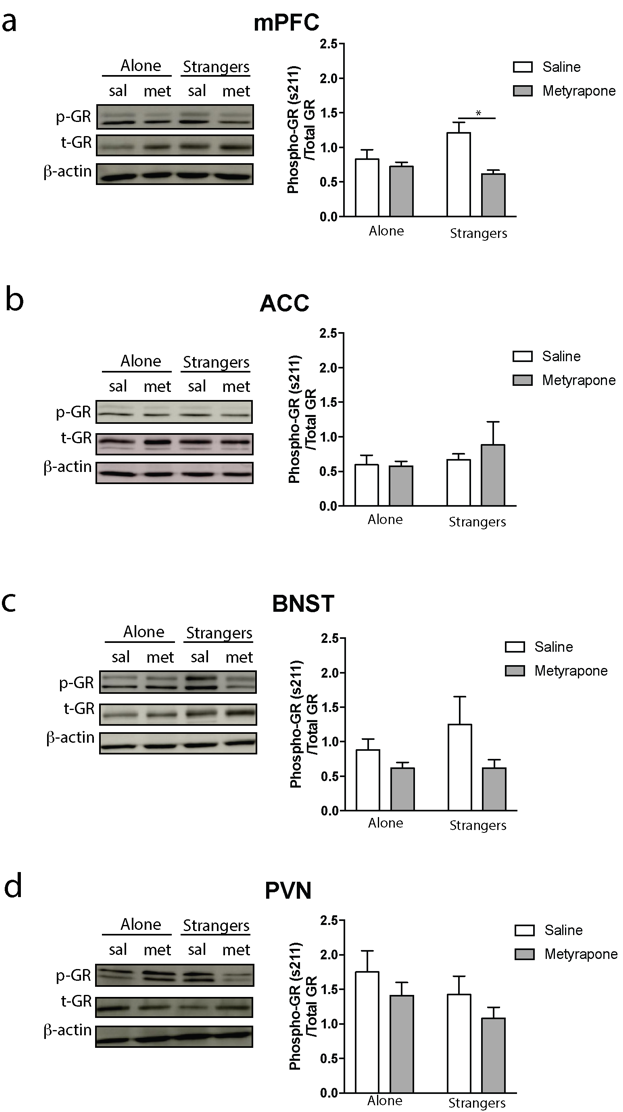


**Supplementary Figure 4**. Western blot analysis showing phosphorylated glucocorticoid (p-GR) receptor expression in brain regions associated with contagion and stress for mice tested alone or in stranger dyads and treated with either vehicle or metyrapone. **a** Increased p-GR expression in the medial prefrontal (mPFC) of stranger dyads following co-writhing behavior is reversed by pre-treatment with metyrapone (50 mg/kg) (two-way ANOVA, *main effect of social context: F_1,20_*=2.52, *p*>0.05; *main effect of drug:* F*_1,20_*=3.32, *p*=0.08; *social context x drug interaction*: F*_1,20_*=4.75, *p*=0.03). **b** p-GR expression in the anterior cingulate cortex (ACC) is not different between the conditions (two-way ANOVA, *main effect of social context: F_1,20_*=0.002, *p*>0.05; *main effect of drug: F_1,20_*=0.85, *p*>0.05; *social context x drug interaction*: *F_1,20_*=0.01, *p*>0.05). **c** Metyrapone pre-treatment reduced overall p-GR expression irrespective of social condition in the bed nucleus of the stria terminalis (BNST) (two-way ANOVA, *main effect of social context: F_1,20_*=3.21, *p*=0.08; *main effect of drug: F_1,20_*=13.74, *p*=0.002; *social context x drug interaction*: *F_1,20_*=1.01, p>0.05). **d** p-GR expression was higher in the paraventricular nucleus (PVN) of the hypothalamus in mice tested alone compared with stranger dyads (two-way ANOVA, *main effect of social context: F_1,20_*=6.47, *p*=0.02; *main effect of drug:* F*_1,20_*=2.95, *p*=0.002; *social context x drug interaction*: *F_1,20_*=2.4, *p*=0.12). *n*=6/condition, bars represent mean ± S.E.M. * *p*<0.05.


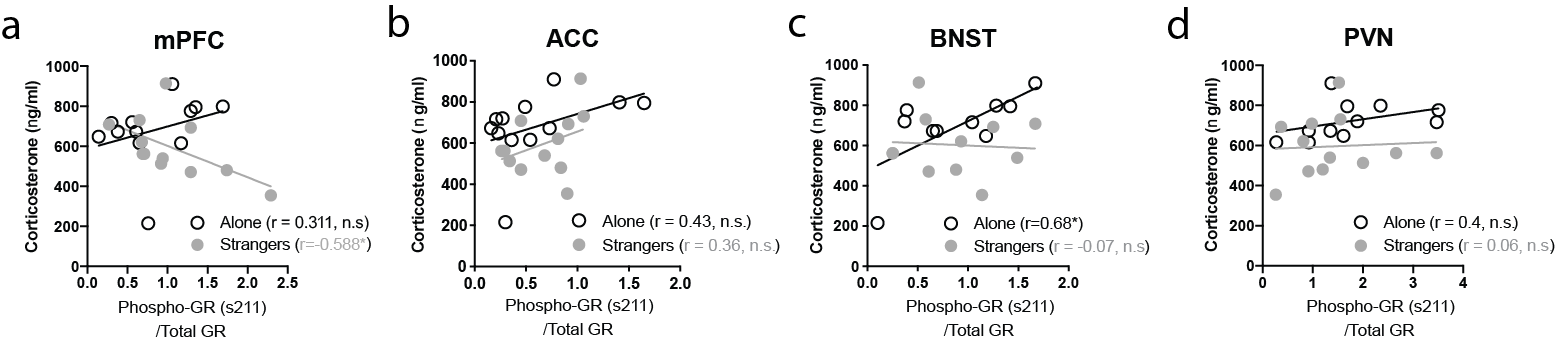


**Supplementary Figure 5**. Association between blood plasma corticosterone (ng/ml) and phosphorylated glucocorticoid receptor (p-GR) expression in brain regions associated with contagion and stress. **a** p-GR expression in the medial prefrontal cortex (mPFC) is negatively correlated with blood plasma corticosterone in stranger dyads, but not mice tested alone (*alone:* *r*=.311, *p*>0.05; *strangers:* *r*=-0.588, *p*<0.05). **b** No significant correlation between p-GR expression in the anterior cingulate cortex (ACC) and blood plasma corticosterone in stranger dyads or mice tested alone (alone: *r*=.43; stranger: 0.36, *p*>0.05) **c** p-GR expression in the bed nucleus of the stria terminalis (BNST) and blood plasma corticosterone is correlated in mice tested along, but not in stranger dyads (*alone:* *r*=0.68, *p*<0.05; *stranger:* *r*=-0.07, *p*>0.05) **d** No significant correlation between p-GR expression in the paraventricular nucleus (PVN) of the hypothalamus and blood plasma corticosterone in stranger dyads or mice tested alone (*alone: r* =0.4, *p*>0.05; strangers: *r*=0.06, *p*>0.06). For all analyses, mice treated with vehicle and metyrapone were included in both the alone and stranger conditions. **p*<0.05.


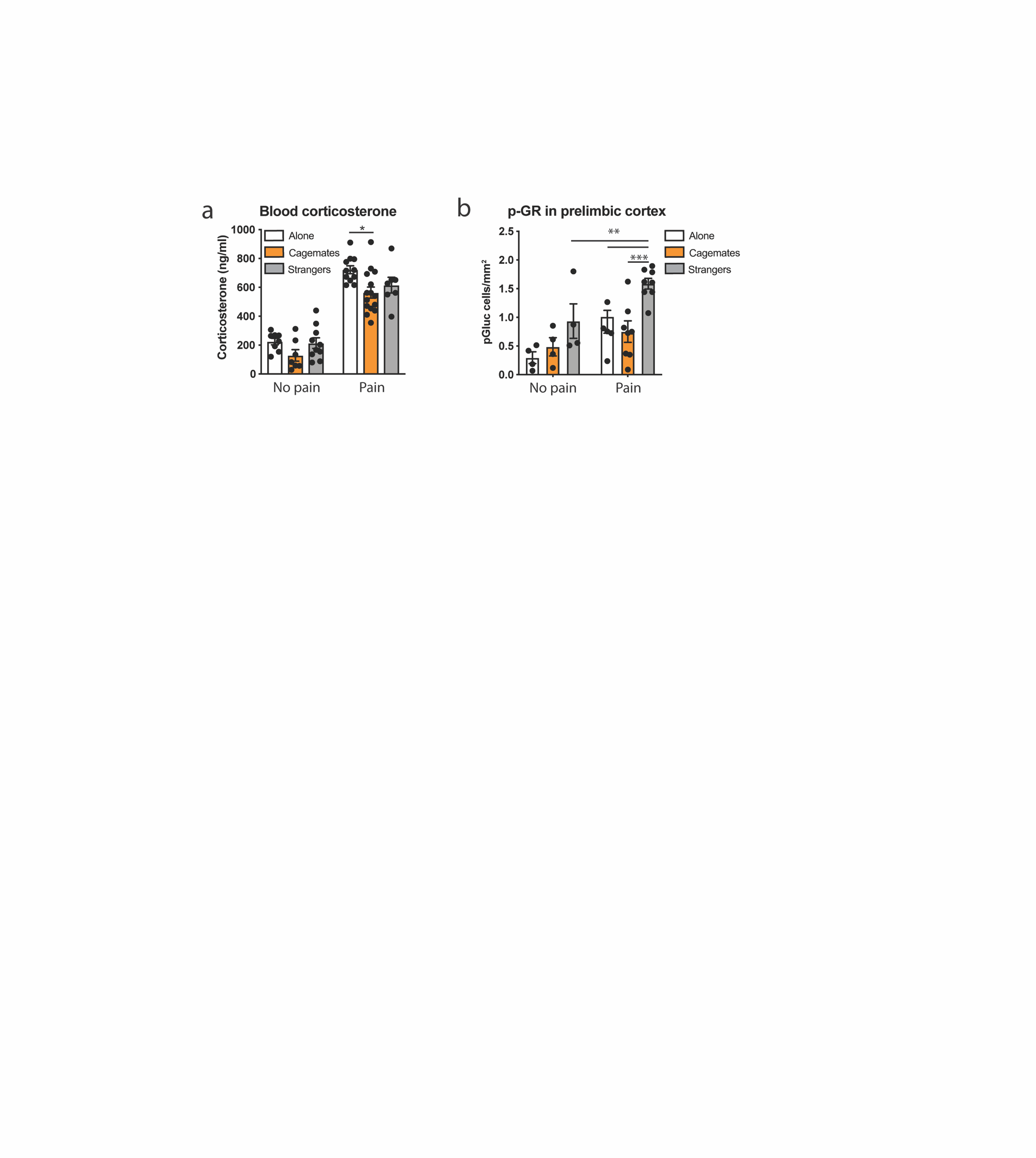


**Supplementary Figure 6.** Measures of stress during the social interaction in the absence (No pain, saline injection) or presence (acetic acid) of a pain stimulus. **a** Plasma blood corticosterone (ng/ml) is elevated during the acetic acid test in all social conditions, but most prominently in mice tested alone (two-way ANOVA, *main effect of social context:* F*_2,52_*=5.44, *p*<0.05; *main effect of pain stimulus*: F*_1,52_*=190.7, *p*<0.0001; *social context x pain stimulus interaction*: F*_2,52_*=0.74, *p*>0.05). **b** Phosphorylated glucocorticoid expression is increased in stranger dyads relative to the other conditions only in the presence of a pain stimulus (two-way ANOVA, *main effect of social*: F*_2,24_*=9.85, p<0.05; *main effect of pain stimulus*: F*_1,24_*=17.79, *p*<0.0001; *social x pain stimulus interaction:* F*_2,24_*=4.063, *p*<0.05). *p<0.05, **p<0.01, ***p<0.001.


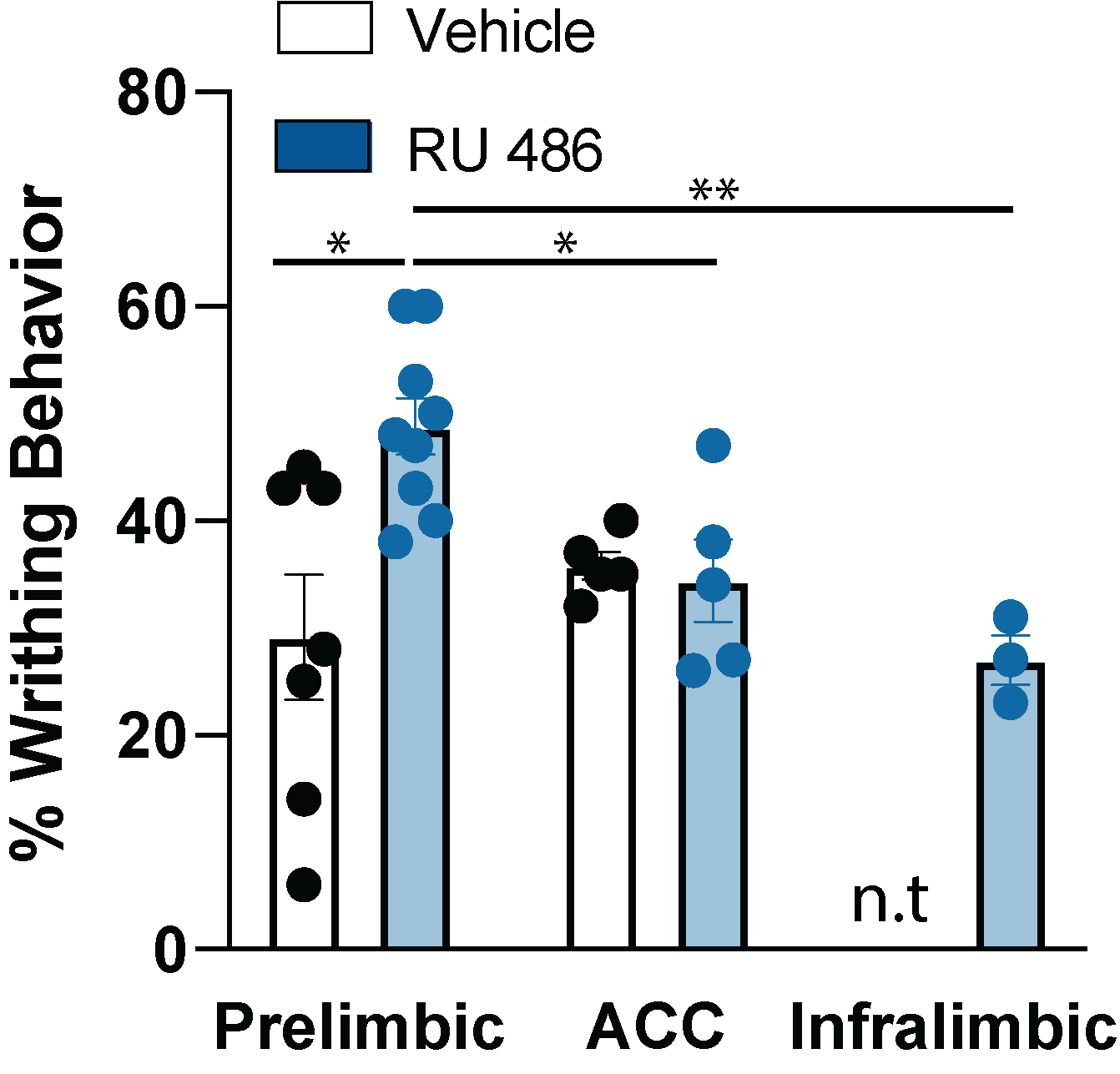


**Supplementary Figure 7.** Data showing microinjections into the infralimbic. Infusions of RU-486 into the infralimbic cortex (n=3 due to cannula misplacement) did not change writhing behavior in stranger dyads. Writhing behavior was increased in stranger mice injected with RU-486 in the prelimbic, but not the ACC or infralimbic (one-way ANOVA, comparing only RU-486 groups, F*_2,14_*=11.36, *p*=0.001). Data from the prelimbic and anterior cingulate cortex (ACC) is replotted from Figure 3. RU-486 bilateral injection into the prelimbic, but not ACC enhances writhing behavior relative to vehicle injected control mice (*prelimbic*: unpaired t-test, t_14_=3.327, *p*=0.005; ACC: unpaired t-test, t_8_=0.3, p>0.05). **p*<0.05, ***p*<0.01.


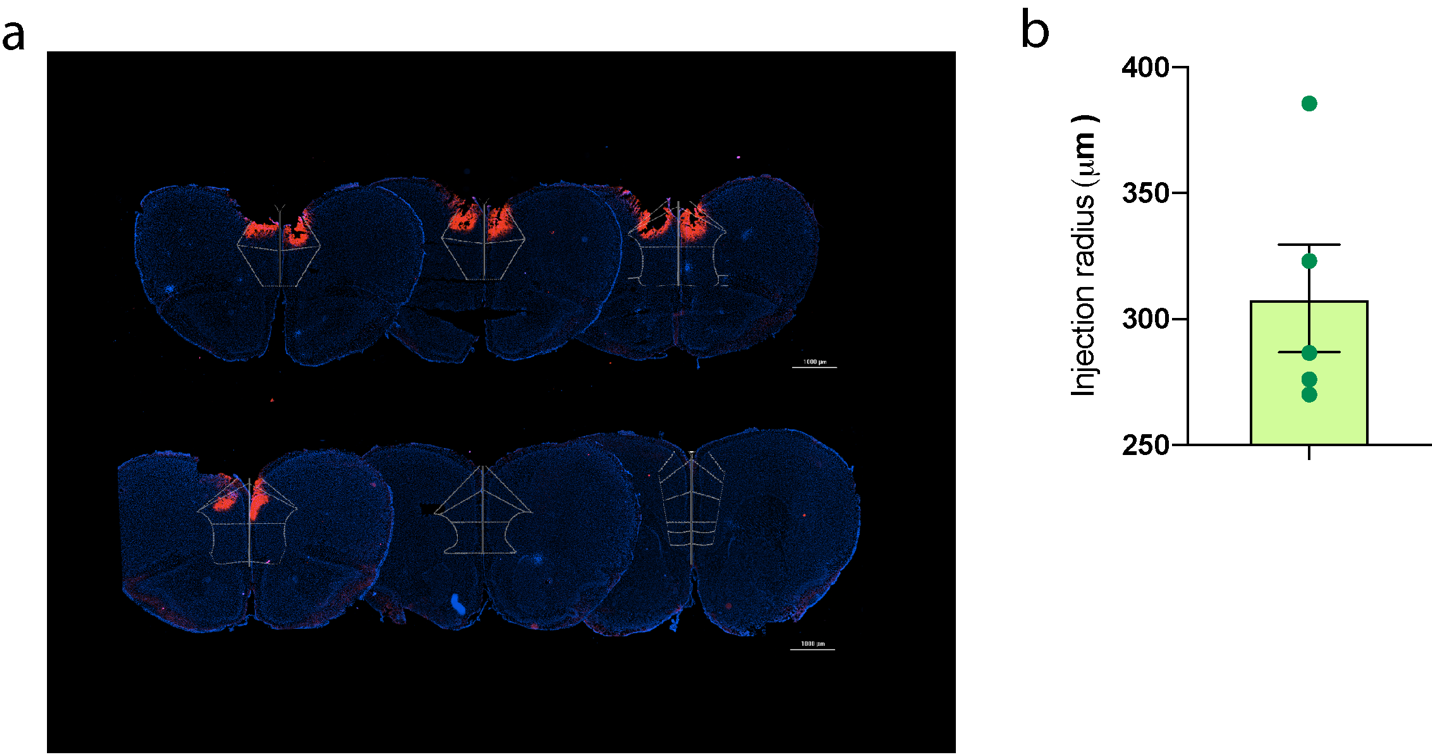


**Supplementary Figure 8.** Prelimbic microinjection (AP1 2.33 mm, ML 0.40 mm, and DV 1.30 mm) of fluorescently labeled muscimol. (0.25 ul per side over 5 min) allowed the visualization of injection spread. **a** Representative image for a muscimol prelimbic microinjection showing minimal cross over to the ACC. **b** The average radius of spread for the fluorescent dye was calculated to be 308.2 ± 21.4 μm using the same injection coordinates, rate and volume as we did for the GR ligands. In order to extrapolate these results to the experiments with the GR ligands that were not labeled with a fluorescent marker (i.e RU-486 and corticosterone), we mapped out all injection sites indicated through histology, and mapped the average radius of spread. Since the exact same injection rate and volume were used, this allowed us to map out which injections were contained to the prelimbic and which to adjacent regions such as the ACC and IL cortex. The average spread of microinjections does not cross into the ACC (approximately 350 μm from the prelimbic injection site).


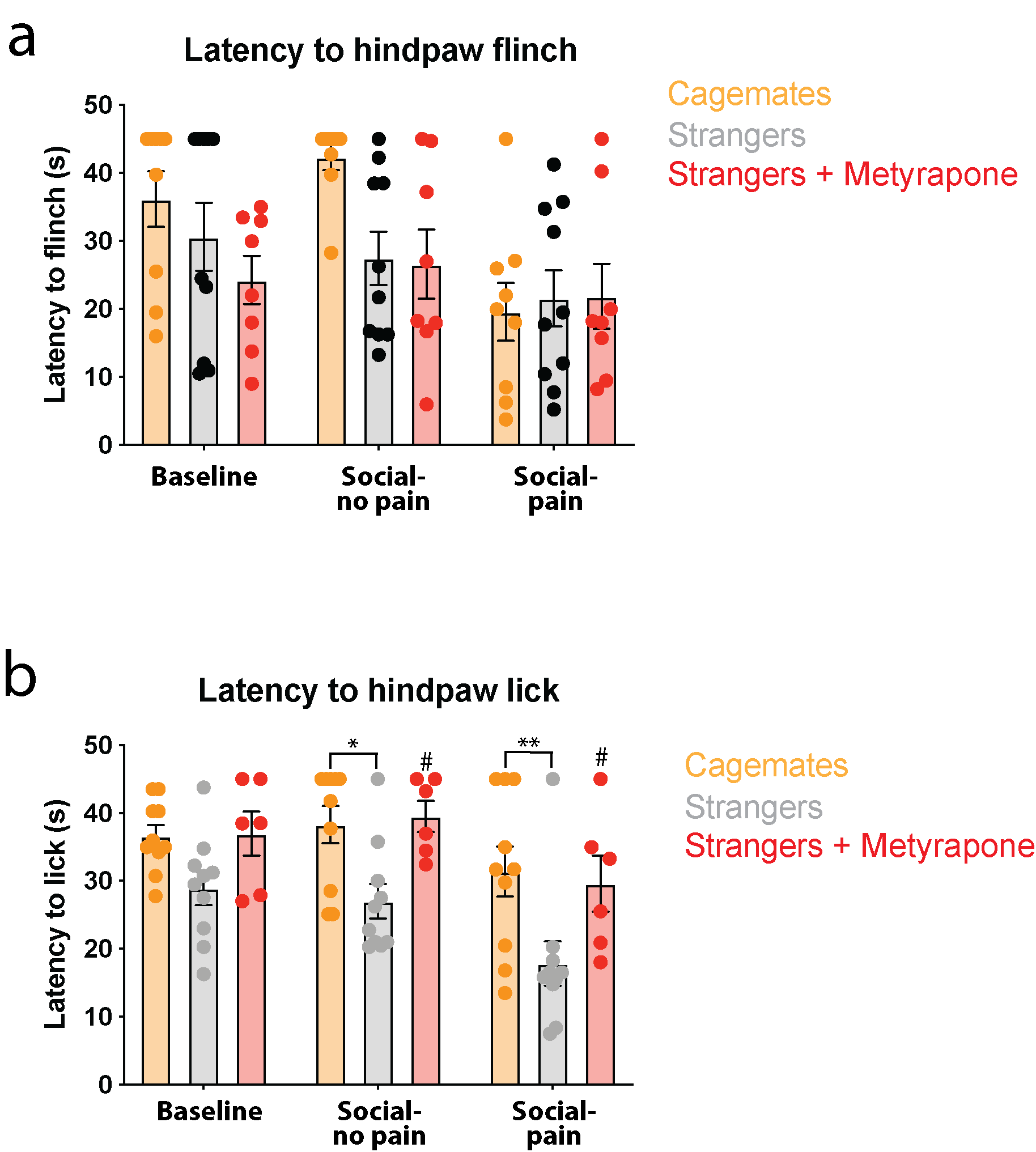


**Supplementary Figure 9.** Latency to first hindpaw flinch or lick on the hotplate during *baseline*, *social-no pain* and *social-pain*. **a** The latency to flinch was not significantly different between any of the dyadic conditions when compared during each phase of testing, however there was a main effect of phase (two-way ANOVA, *main effect of social context:* *F_2,25_*=2.64, *p*=0.09; *main effect of phase (RM):* *F_2,50_*=6.71, *p*<0.05; *social context x phase of testing:* *F_4,50_*=1.80, *p*>0.05). **b** Latency to the first observed hind paw lick is reduced in stranger dyads following a 30 min social interaction (*social-no pain*) and following acetic acid injection (*social-pain*). At both phases of testing, metyrapone (50 mg/kg) injected strangers were approaching significance when compared with stranger dyads (two way ANOVA, *main effect of social context:* *F_2,25_*=10.96, *p*<0.001; *main effect of phase (RM):* *F_2,50_*=8.79, *p*<0.001; *social context x phase of testing: F_4,50_*=0.41, *p*>0.05).**p* < 0.05, ***p* < 0.001 compared within testing phase (baseline, social-no pain, social-pain) by Tukey’s post hoc test. #*p*=0.06 compared with analogous stranger dyad.
